# Temperature sensitive nanogels for real-time imaging during transcatheter arterial embolization

**DOI:** 10.1080/15685551.2022.2164445

**Published:** 2023-01-18

**Authors:** Hongfu Zhou, Wenjing Xie, Anran Guo, Bin Chen, Sanming Hu, Min Zheng, Houqiang Yu, Hongan Tian, Ling Li

**Affiliations:** a School of Biomedical Engineering and Imaging, Xianning Medical College, Hubei University of Science and Technology, Xianning, PR China; b Department of Radiology, Xianning Central Hospital, the First Affiliated Hospital of Hubei University of Science and Technology, Xianning, P.R. China; c School of Pharmacy, Xianning Medical College, Hubei University of Science and Technology, Xianning, PR China; d Department of Mathematics and Statistics, Hubei University of Science and Technology, Xianning, PR China

**Keywords:** Embolization, X-ray visible, high-strength nanogels, real-time imaging, temperature-sensitive

## Abstract

Several vascular embolization materials are commonly used in clinical practice, however, having application defects of varying degrees, such as poor intraoperative imaging and easy recanalization of embolized blood vessels, they are challenging for application during Transcatheter arterial embolization (TAE). Thus, an intraoperative visible vascular embolization material with good embolization effect and biocompatibility can improve transcatheter arterial embolization clinical efficacy to some extent. Our study aimed to synthesize a novel vascular embolization material that can achieve complete embolization of arterial trunks and peripheral vessels, namely poly (N-isopropyl acrylamide)-*co*-acrylic acid nanogel (NIPAM-*co*-AA). Iohexol 200 mg/mL was co-assembled with 7 wt% NIPAM-*co*-AA nanogel to create an intelligent thermosensitive radiopaque nanogel (INCA), which achieves a good intraoperative imaging effect and is convenient for transcatheter arterial bolus injection due to its good fluidity and temperature-sensitive sol-gel phase transition. The normal rabbit kidney embolism model further confirmed that INCA could effectively use Digital subtraction angiography (DSA) to achieve intraoperative imaging, and real-time monitoring of the embolization process could avoid mis-embolization and leakage. Meanwhile, in a 42-day study, INCA demonstrated an excellent embolization effect on the right renal artery of New Zealand white rabbits, with no vascular recanalization and ischemic necrosis and calcification remaining. As a result, this radiopaque thermosensitive nanogel has the potential to be an intelligent thermosensitive medical vascular embolization material, providing dual benefits in TAE intraoperative imaging and long-term postoperative embolization while effectively addressing the shortcomings and challenges of commonly used clinical vascular embolization agents.

## 1. Introduction

Embolization, an endovascular therapy primarily involving the introduction of embolic materials into arteries to embolize the arteries and capillaries that feed tumors, is a widely accepted palliative treatment for cancers because of its low risk and high success rate [1–4]. Several embolic materials are currently available in clinics, including polyvinyl alcohol (PVA) microparticles, lipiodol, and ethylene (vinyl alcohol) polymer and precipitant gels [5–7]. Because of their fluidity, solid embolic agents come in a variety of sizes and shapes to match the size and caliber of the target vessel [8–11]. Moreover, there is growing interest in liquid embolic agents because they can be delivered quickly through a microcatheter, permeate and fill the target vessels, and eventually form a complete occlusion independent of the patient’s coagulation system [12–14].

Common liquid embolic agents include sclerosing agents, lipiodol, cyanoacrylate glues, and Onyx [15–17], which use different strategies to occlude vessels. Ethanol, as an example of a sclerosing agent, damages endothelial cells and denatures blood proteins, resulting in blood vessel necrosis and sclerosis. Lipiodol is widely used in Transcatheter arterial chemo-embolization (TACE) clinical therapy because it can quickly cut off the tumor blood supply [18–20]. Achieving long-term embolization of tumor arteries is challenging due to blood scouring and rapid in-vivo elimination [21–23]. Although current liquid embolic agents can most clinical needs, they have some limitations [24,25]. As a result, novel liquid embolic agents are being developed in order to improve imaging properties, reduce complications, and optimize flow performance [26–28].

**Scheme 1.**
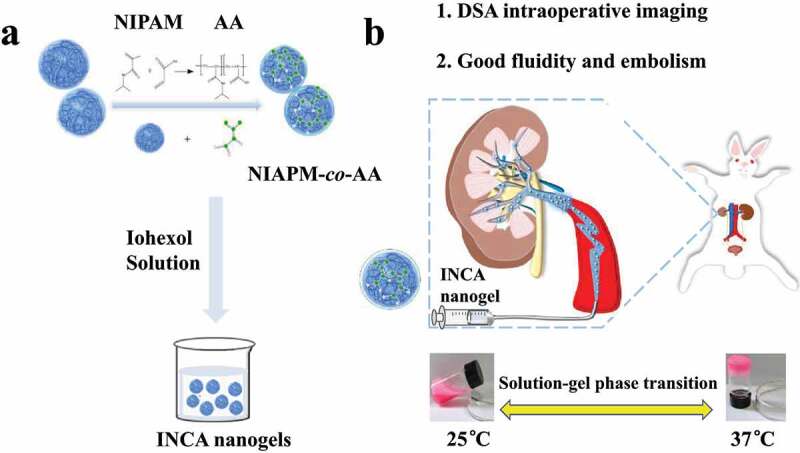
Schematic diagram of smart thermosensitive X-ray radiopaque INCA nanogel with intraoperative visualization and durable embolization properties in rabbit renal arteries.

Our study used 2,2’-azobis(2-methylpropionamidine) dihydrochloride with a better initiation effect, a water-soluble azo initiator that can carry out a smooth, stable, and controllable decomposition reaction (Scheme 1a). Without nitrogen treatment, it only needed to continue the reaction at the initiation temperature. Simultaneously, it was combined with the cationic emulsifier 2,2’-azobis (2- methyl propamidine dihydrochloride). The polymerization process of N-isopropyl acrylamide and acrylic acid had a better emulsification effect, which aided in the formation of polymer hydrogels with stable systems. A poly (N-isopropyl acrylamide)-*co*-acrylic acid hydrogel (NIPAM-*co*-AA) was synthesized easily and efficiently without the need for additional salt and pH adjustments, and the nanogel could be directly induced to gelation at 34°C, significantly improving the convenience and achievability of clinical translational applications.

Iohexol is a non-ionic X-contrast agent raw material which, after mixing NIPAM-*co*-AA nanogel (7 wt%) with a concentration of 200 mg/mL, has an X-ray effect similar to iodized oil. The radiopaque NIPAM-*co*-AA nanogel (INCA) with synergistic iohexol X-contrast agent has excellent arterial vascular embolization and provides convenient real-time monitoring of images. In the normal rabbit renal artery embolization model, INCA nanogel could achieve complete embolization of the small blood vessels of the main trunk and periphery of the right renal artery of the rabbit. During the embolization treatment for up to 42 days, the embolization site did not appear to have vascular recanalization and establishment of collateral circulation. Due to insufficient blood supply, the right kidney of rabbits showed obvious ischemic necrosis and calcification (Scheme 1b). This showed that this smart temperature-sensitive nanogel embolization material with X-ray radiopaque has good advantages in intraoperative imaging and long-term postoperative embolization, as well as potential in the future clinical treatment of TAE.

## 2. Materials and methods

### 2.1. Materials and animals

N-isopropyl acrylamide (NIPAM), acrylic acid (AA), and iohexol were purchased from Tokyo Co., Ltd., Japan. N, N’-methylene bisacrylamide (MBA), octadecyl trimethyl ammonium chloride (STAC), 2,2’-azobis(2-methylpropionamidine) dihydrochloride (AAPH), and rhodamine-B were purchased from Aladdin Chemical Reagent Co., Ltd. Pentobarbital sodium, and 4% paraformaldehyde were purchased from McLean Reagent Co., Ltd. Iodized oil and iodixanol were purchased from Jiangsu Hengrui Pharmaceutical Co., Ltd. In contrast, polyvinyl alcohol was purchased from Hangzhou Ailikang Pharmaceutical Technology Co., Ltd. Lidocaine hydrochloride, gentamicin sulfate, and penicillin sodium were purchased from North China Pharmaceutical Co., Ltd. Heparin sodium injection was purchased from Changzhou Qianhong Biochemical Pharmaceutical Co., Ltd. sodium chloride injection (0.9%) was purchased from Wuhan Binhu Shuanghe Pharmaceutical Co., Ltd. Milli-Q ultrapure water (18.2 MΩ) was used for all experiments.

Adult New Zealand white rabbits (body weight: 2.5–3.0 kg, hermaphrodite) were purchased from the Laboratory Animal Center of Hubei University of Science and Technology, and all animal procedures were performed in accordance with the Guidelines for Care and Use of Laboratory Animals of the Science and Technology Department (Hubei Province, China) and approved by the Animal Ethics Committee of Hubei University of Science and Technology.

### 2.2. Synthesis of NIPAM-co-AA nanogels

First, the formula group design was carried out by optimizing and improving the emulsion polymerization method. The optimal ratio of hydrogel was screened out, and a NIPAM-*co*-AA nanogel composed of amino groups was efficiently synthesized (Table S1). Briefly, NIPAM (1.7 g, 15 mmol), MBA (0.03 g, 0.19 mmol), STAC (0.05 g, 0.29 mmol), AAPH (0.08 mg, 0.14 mmol) was dissolved in 150 mL of ultrapure water and continuously heated to 75°C in the three-necked flask. The reaction was stirred at a constant temperature and 200 rpm/min until the solution was initiated completely. After the solution turned blue, AA (215 µL, 3 mmol) was added quickly to the reaction solution. When the solution gradually changed from light blue to milky white, rhodamine B (0.02 g, 0.04 mol) was added, and the reaction was allowed to run for 4 hours before being stopped. The collected nanogel aqueous solution was dialyzed for 3–4 days in ultra-pure water using a dialysis bag (molecular weight cut-off point: 14,000 Da), and the freeze-dried nanogel powder was collected for future use.

### 2.3. Preparation of INCA nanogels composite dispersion system

Iohexol was formulated at concentrations of 40 mg I/mL, 80 mg I/mL, 120 mg I/mL, 160 mg I/mL, and 200 mg I/mL using Milli-Q ultrapure water. The concentrations of NIPAM-*co*-AA nanogels were weighed and mixed with different iohexol solutions (2 mL) to make radiopaque INCA nanogels and dispensed into transparent glass bottles.

### 2.4. Characterization

The freeze-dried nanogels were mixed with potassium bromide, ground, pressed into tablets, and scanned 16 times in an infrared spectrometer with a resolution of 4 cm^−1^ for nanometers (Platinum Elmer Inc., USA). The infrared-absorbing functional groups in nanogels were investigated. The particle size and dispersion of the nanogels were evaluated using a He-Ne laser source (λ = 633 nm, scattering angle: 90°) and dynamic light scattering (Zetasizer Nano ZS90, Malvern Instruments Ltd., Malvern, UK). The nanogels samples morphology was observed after the freeze-dried nanogels powder was sprayed with gold at 20 mA for 60s, and the acceleration voltage was 5 kV. A drop of nanogels dispersion (0.05 wt%) was placed on the copper mesh containing carbon support film and dried at 25°C for 10 min. Phosphotungstic acid solution (0.1 wt%) was used to stain, rinsed with PBS, and dried. The morphology of the nanogels was characterized using a transmission electron microscope (Tecnai G2 20, FEI Corp., Eindhoven, The Netherlands) at 200 kV.

One drop of the diluted nanogel aqueous dispersion was placed on the mica sheet and observed and photographed using an atomic force microscope AFM (DI Nanoscope IV, Veeco, USA) after drying. The viscoelasticity of the nanogels dispersion was measured using a high-speed rotational rheometer (Malvern Instruments Ltd., Malvern, UK), with the following parameters: PP50 plate, diameter 50 mm, gap 0.5 mm, pressure 0.05 Pa, temperature range 30 °C ~ 50 °C, a heating rate 1.0 °C/min, frequency 1.0 Hz.

A UV-visible spectrophotometer (Beijing General Analytical Instrument Co., LTD., China) was used to measure the change in light transmittance at 500 nm with a 1 wt% concentration at a heating rate of 1 °C/min in the temperature range of 20°C to 50°C. The nanogels aqueous dispersion with a concentration of 4 wt % was used to characterize the temperature sensitivity of the nanogel in the range of 25°C to 45°C using a differential thermal scanner (DSC) at 0.5 °C/min. X-ray attenuation was assessed on NIPAM-*co*-AA nanogels with different concentration iohexol solutions using computed tomography (SOMATOM Sensation 64 Spiral; Siemens) in body PCT mode at 100 kV and 80 mA.

### 2.5. Evaluation of renal artery embolization in normal rabbits using INCA nanogels

First, 13 normal New Zealand rabbits were divided randomly into four groups (3 in each group), and the remaining was sacrificed immediately after renal embolization with rhodamine-B labeled INCA nanogels. After fasting for 12 h, pentobarbital sodium (30 mg/kg) was injected through the auricle vein of rabbits, and fixed supine after 5–15 min when pain disappeared. The skin of the groin was shaved and disinfected, and the femoral artery was separated with eye forceps. An 18 G puncture needle introduced a 4 F coaxial microcatheter (Terumo, Tokyo, Japan) into the proximal artery. Then the contrast agent iodixanol (300 mg I/mL, 0.5 mL/s) was injected to perform angiography of the renal artery to determine whether the blood vessels were good and patency. INCA nanogels (1.5 mL), PVA, and iodized oil were administered into normal rabbits’ right renal arteries, respectively. The entry of embolization into the renal artery at 1s, 3s, 5s, 8s, and 10s after injection was observed in real-time through digital subtraction angiography (DSA; Siemens BICORTOP, Germany), and the rabbits in each experimental group were followed up with a CT at 7 d, 21 d and 42 d after embolization, respectively, to observe the effect of renal artery embolization and recanalized vessels. After 42 days of treatment, the rabbits were euthanized, and the liver, spleen, lung, and kidney tissues were subjected to H&E and Masson staining to analyze the pathological changes after embolization.

### 2.6. Biocompatibility evaluation

New Zealand rabbit whole blood (2 mL) was taken for the hemolysis experiment. Briefly, the red blood cells were collected by centrifugation (1,500 rpm × 10 min) by mixing whole rabbit blood and normal saline (1,500 rpm × 10 min) until the supernatant became colorless and prepared into a 2% red blood cell suspension. The dilution concentrations were prepared 120 µg/mL, 60 µg/mL, 30 µg/mL, 15 µg/mL, and 7.5 µg/mL, respectively, taking 7 wt% INCA nanogels dispersion and 200 mg/mL iohexol solution as the test group. Normal saline was used as the negative control group, and ultrapure water as the positive control group.

A blood compatibility test was carried out five times in each group. The solution (300 µL) from each group was pipetted into a 1.5 mL EP tube, and 300 µL red blood cell suspension was added, incubated at constant temperature for 1 h, centrifuged at 3000 rpm for 25 min, and 100 µL of the supernatant was added to a 96-well plate. The OD value was measured using a microplate reader (λ = 540 nm; 1420 Multilabel Counter, PerkinElmer), and the hemolysis rate (Hr %) was calculated as:

$$Hr\%\frac{OD_{S}-OD_{n}}{OD_{b}-OD_{n}}\times 100\%$$

OD_s_ represents the absorbance of the test group, OD_b_ represents the absorbance of the blank group, and OD_n_ represents the absorbance of the negative control group.

The cytotoxicity test of HepG2 cells was carried out by the MTT method. After HepG2 cells were cultured to the logarithmic growth phase, the cells were digested and collected. The cells were diluted to 1 × 10^5^ cells /mL and plated in 96-well plates at 5 × 10^3^ per well. After culturing the cells for 24 h, the medium was aspirated. The 7 wt% INCA nanogel dispersion and 200 mg/mL iohexol solution were diluted to 560 µg/mL, 280 µg/mL, 140 µg/mL, 70 µg/mL, and 35 µg/mL, respectively, and added to DMEM medium for incubation with HepG2 cells for 24 h. MTT (20 µL) with a concentration of 5.0 mg/mL was added and incubated for 4 h, followed by 150 µL DMSO. After 20 min, the absorbance at 490 nm was recorded using a microplate reader, and cell viability was calculated according to the formula.

$$CV,\%\frac{OD_{s}-OD_{b}}{OD_{n}-OD_{b}}\times 100\%$$

OD_s_, OD_b,_ and OD_n_ represent the OD values of the sample, blank, and negative control group, respectively.

On the 5th, 10th, 15th, and 20th days after TAE treatment, the body weight of the experimental rabbits in each group were measured, and the relative body weight (RBW %) was calculated using the formula:

$$RBW\%\frac{W_{t}-W_{0}}{W_{o}}\times 100$$

W_o_ and W_t_ represent the body weight of experimental rabbits on the day ‘t’ before and after TAE treatment, respectively.

## 3. Results and discussion

### 3.1. *Structure and morphology characterization of NIPAM-*co*-AA nanogels*

**Figure 1.**
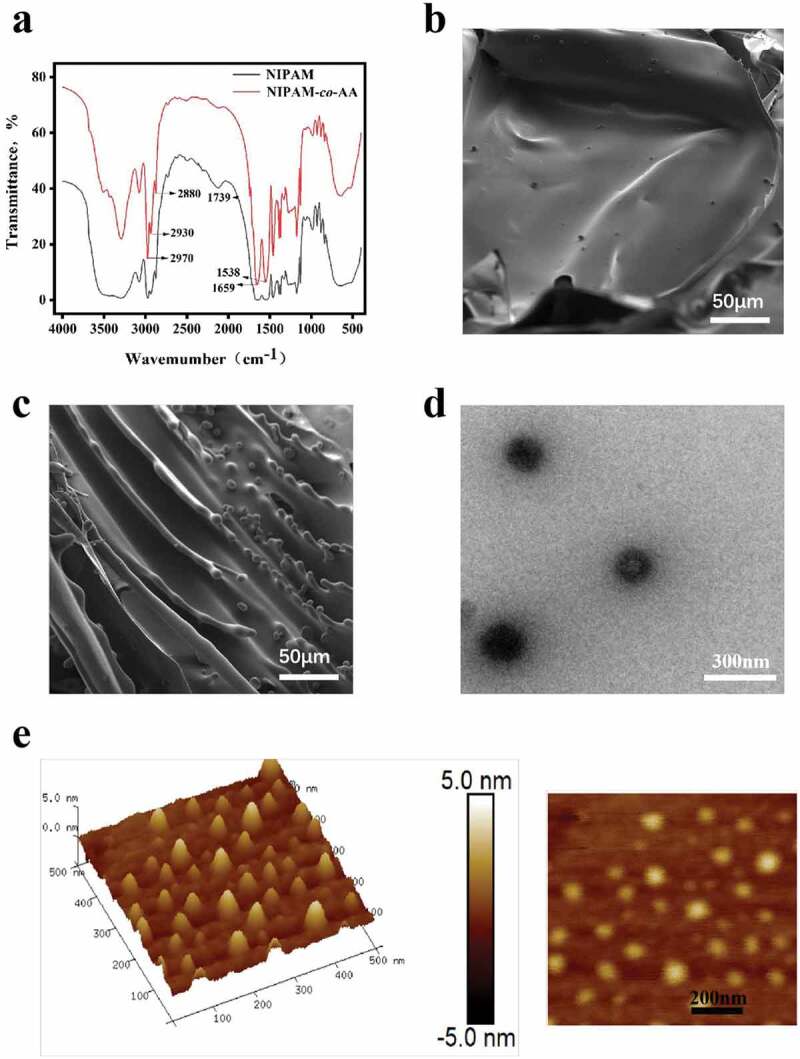
Structure and morphology of NIPAM-*co*-AA nanogels. (a) The IR-absorbing functional groups of NIPAM (solid black line) and NIPAM-*co*-AA nanogels (solid red line) measured by near-infrared spectroscopy. (b) and (c) SEM image of NIPAM and NIPAM-*co*-AA nanogels with a scale of 50 µm. (d) TEM image with a scale of 300 nm. (e) Atomic mechanical micrograph of NIPAM-*co*-AA nanogels.

Our study developed temperature-sensitive NIPAM-*co*-AA nanogels as an intelligent embolism material to solve most embolization materials flow problems. The infrared absorption functional groups of the NIPAM-*co*-AA nanogels were analyzed using an infrared spectrometer. Figure 1(a) shows C-H stretching vibration peaks of methyl and methylene appeared at 2970 cm^–1^, 2930 cm^–1^, and 2880 cm^–1^, N-H bending vibration peaks of amide I (-NH I) and amide II (-NH II) appeared at 1659 cm^–1^ and 1538 cm^–1^, and the carboxyl group in acrylic acid appeared at 1739 cm^–1^, indicating that acrylic acid was successfully grafted into the hydrogel system.

SEM images revealed that the micromorphology distribution of NIPAM nanogels was uniform and flat, whereas that of NIPAM-*co*-AA nanogel after polymerization with acrylic acid changed correspondingly, displaying a hump-like bump in Figure 1(b, c). TEM and atomic force microscopy were also used to characterize the morphology and structure of NIPAM-*co*-AA nanogels, and a spherical nanostructure was discovered in Figures 1(d, e).

### 3.2. *Characterization of temperature-sensitive properties of NIPAM-*co*-AA nanogels*

**Figure 2.**
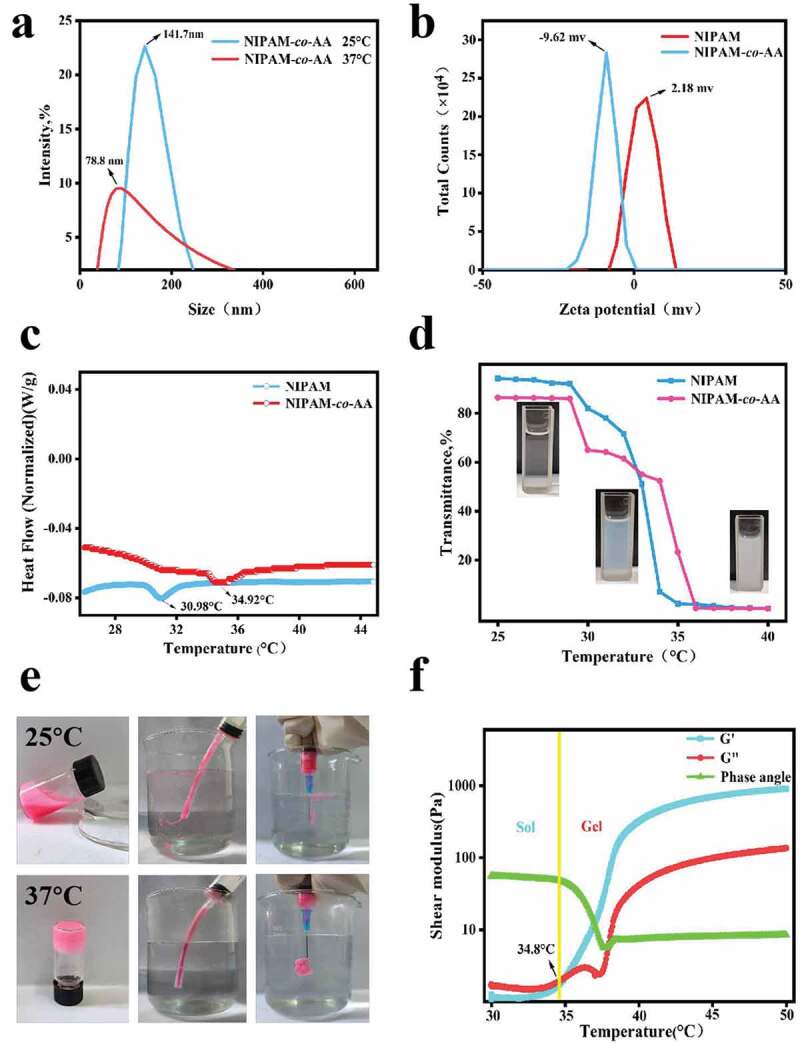
Characterization of temperature-sensitive properties of NIPAM-*co*-AA nanogels. (a) The particle size change of NIPAM-*co*-AA nanogels at 25°C and 37°C analyzed by a dynamic light scattering particle size analyzer. (b) Zeta potential changes of NIPAM and NIPAM-*co*-AA nanogels. (c) Comparison of differential thermal scanning analysis of NIPAM and NIPAM-*co-*AA nanogels. (d) The transmittance changes of NIPAM and NIPAM-*co*-AA nanogels measured by UV-VIS spectrophotometer with increasing temperatures from 25 °C ~ 40 °C. (e) The sol-gel phase transition of NIPAM-*co*-AA nanogels stained with Rhodamine-B at 25°C and 37°C observed by bottle reversal visual method and injection through catheter and syringe. (f) The G ‘and G ”moduli of NIPAM-Co-AA nanogels measured between 30°C and 50°C using a high-speed rotary rheometer.

The average particle size and dispersion of 0.2 wt% NIPAM-co-AA at 25°C and 37°C were analyzed using a dynamic light scattering particle size analyzer (Figure 2a). The results showed that the average particle size of NIPAM-*co*-AA nanogels at 25°C was concentrated at 141.7 nm, and the PDI was 0.252. The average particle size of NIPAM-*co*-AA nanogels at 37°C was concentrated around 78.8 nm, and the PDI was 0.236, indicating a strong temperature sensitivity. When the temperature rises from 25°C to 37°C, the size of the nanogels can be reduced by two-thirds. The main reason is that with the increase in temperature, the hydrophobicity of the nanogels increases, that is, the shrinkage state caused by the temperature rise. At the same time, the swelling was inhibited; therefore, the particle size of the nanogels decreased accordingly, the overall particle size was small, and the dispersibility was good at 25°C and 37°C. Furthermore, the Zeta potential results revealed that the Zeta peak position of the NIPAM nanogels without the addition of AA hydrophilic monomer was 2.18 mV. In comparison, the zeta peak position of the NIPAM-*co*-AA nanogels was – 9.62 mV, indicating that the carboxyl group (-COOH) in acrylic acid significantly increased the number of negative charges in the nanogels system (Figure 2b).

The differential thermal scanning analysis revealed that a discontinuous phase transition occurred between NIPAM and NIAPM-*co*-AA nanogel as the temperature increased from 25°C to 45°C, accompanied by an endothermic phenomenon (Figure 2c). The peak position of the enthalpy changed at 30.98°C and 34.92°C, respectively, which is the sol-gel phase transition point. A UV-VIS spectrophotometer was used to measure the transmittance changes of NIPAM and NIPAM-*co*-AA nanogels at 25°C and 40°C (Figure 2d). The water dispersion of the nanogels was initially transparent and gradually changed to light blue until milky white as the temperature increased, and the transmittance continued to decrease. The turbidity point temperatures of NIPAM and NIPAM-*co*-AA nanogels were approximately 31°C and 34°C, respectively, when the light transmittance was reduced to 50%. It is evident from the inverted bottle by comparing the changes in NIPAM-*co*-AA nanogel at 30 °C ~ 36 °C, the sol-gel phase transformation could not be achieved when the concentration was 1 wt% because the gel concentration was too low. In contrast, when the concentration of NIPAM-*co*-AA nanogels was 4 wt% and 7 wt%, gelation could be achieved at 34°C (Figure S1). However, as the temperature of 4 wt% nanogels increased, many water molecules precipitated from the inside of the nanogels. In general, 7 wt% NIPAM-*co*-AA nanogels had better coagulation performance. Subsequently, the 7 wt% NIPAM-*co*-AA nanogels labeled with Rhodamine B were inverted by the bottle to more visually observe the transformation of the soluble gel phase.

The TAE treatment was simulated by syringe and catheter injection at 25°C and 37°C, respectively. At 25°C, NIPAM-*co*-AA nanogels were injected with good fluidity through a syringe and catheter and could be well dispersed in the aqueous medium. However, at 37°C, NIPAM-*co*-AA nanogels solidified in situ in the catheter cavity and could not flow and disperse in the aqueous medium. In addition, the nanogels pushed from the syringe also coagulated rapidly and blocked the pinhole at 37°C (Figure 2e). The elastic modulus (G’) and viscous modulus (G ”) of NIPAM-*co*-AA nanogels were tested using a high-speed rotary rheometer (Figure 2f). A mobile sol phase appeared when the temperature was lower than about 34.8°C (G’ < G”), while when the temperature was higher than 34.8 °C (G’> G”), a non-mobile gel phase appeared. Thus, the solution-gel phase transition point of NIPAM-*co*-AA was near 34.8°C.

### 3.3. *Evaluation of* in-vitro *and* in-vivo *imaging effect of INCA nanogels*

**Figure 3.**
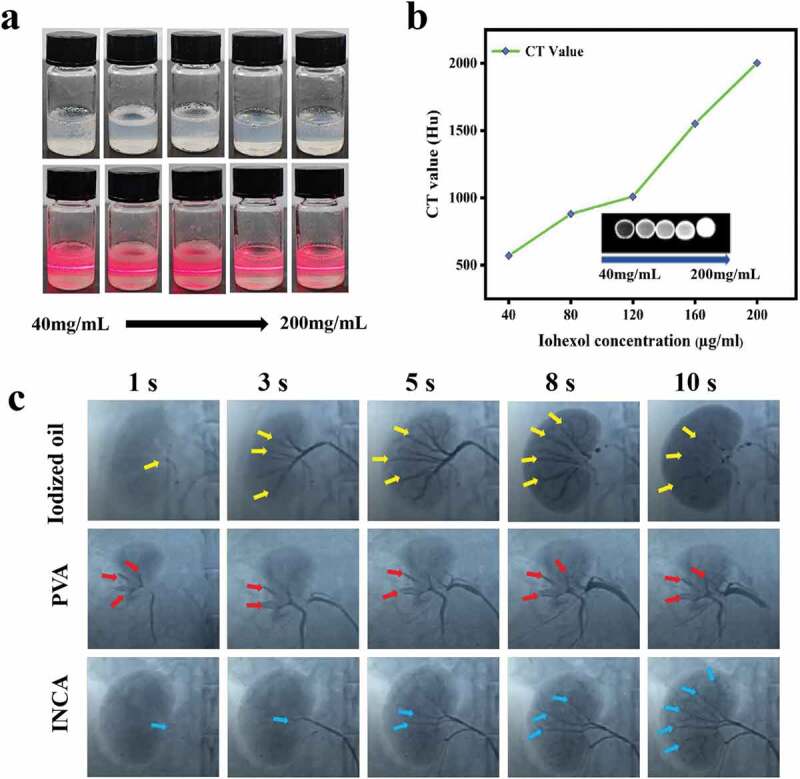
Evaluation of *in-vitro* and *in-vivo* imaging effect of INCA nanogels. (a) Tyndall effect occurred after laser irradiation when NIAPM-*co*-AA nanogels were mixed with iohexol solutions of different concentrations (40 µg/mL, 80 µg/mL, 120 µg/mL, 160 µg/mL, and 200 µg/mL). (b) NIAPM-*co*-AA nanogels were mixed with iohexol solutions containing 40 µg/mL, 80 µg/mL, 120 µg/mL, 160 µg/mL, and 200 µg/mL at different concentrations, and computed tomography was performed to determine INCA nanogels CT values. (c) DSA silhouette of iodized oil, PVA, and INCA nanogels 1s, 3s, 5s, 8s, and 10s after injection into the renal artery.

NIAPM-*co*-AA nanogels (7 wt%) were mixed with different concentrations of iohexol solutions to enhance the visualization of INCA nanogels during embolization *in-vivo*. A bright “pathway” was observed in the nanogels by shining a laser beam vertically on INCA nanogels, mainly due to the Tyndall effect caused by the scattering of light by the colloidal particles (Figure 3a). Subsequently, the imaging effect was measured in-vitro by computed tomography scanning. The CT value was quantitatively analyzed to screen the concentration of iohexol solution suitable for CT imaging follow-up. When iohexol solution at 40 µg/mL, 80 µg/mL, 120 µg/mL, 160 µg/mL, and 200 µg/mL, the corresponding CT values were 571.0 ± 70.09 Hu, 881.0 ± 11.59 Hu, 1010.69 ± 11.38 Hu, 1552.88 ± 80.88 Hu, and 2003.75 ± 19.08 Hu, respectively (Figure 3b), indicating that the imaging effect was best at 200 µg/mL, which could meet the requirements of *in-vivo* CT imaging.

A 4 F microcatheter was used to inject iodized oil, PVA, and INCA nanogel into rabbits’ right renal artery to evaluate intraoperative imaging’s effect further. Figure 3c shows the DSA profiles of 1s, 3s, 5s, 8s, and 10s during the operation. INCA nanogels and iodized oil groups showed similar intraoperative imaging effects during the injection process. Clear renal blood flow was observed, directly reaching the renal aorta, interlobar artery, and arcuate artery, which can avoid missed embolization and mis-embolization and comprehensively improve the embolization effect of TAE. However, PVA can only be used with iodized oil for real-time monitoring of the entire embolization process because of its large size and no imaging effect.

3.4. Evaluation of the embolization effect of INCA nanogels on the blood vessels of normal rabbit renal arteries at all levels

**Figure 4.**
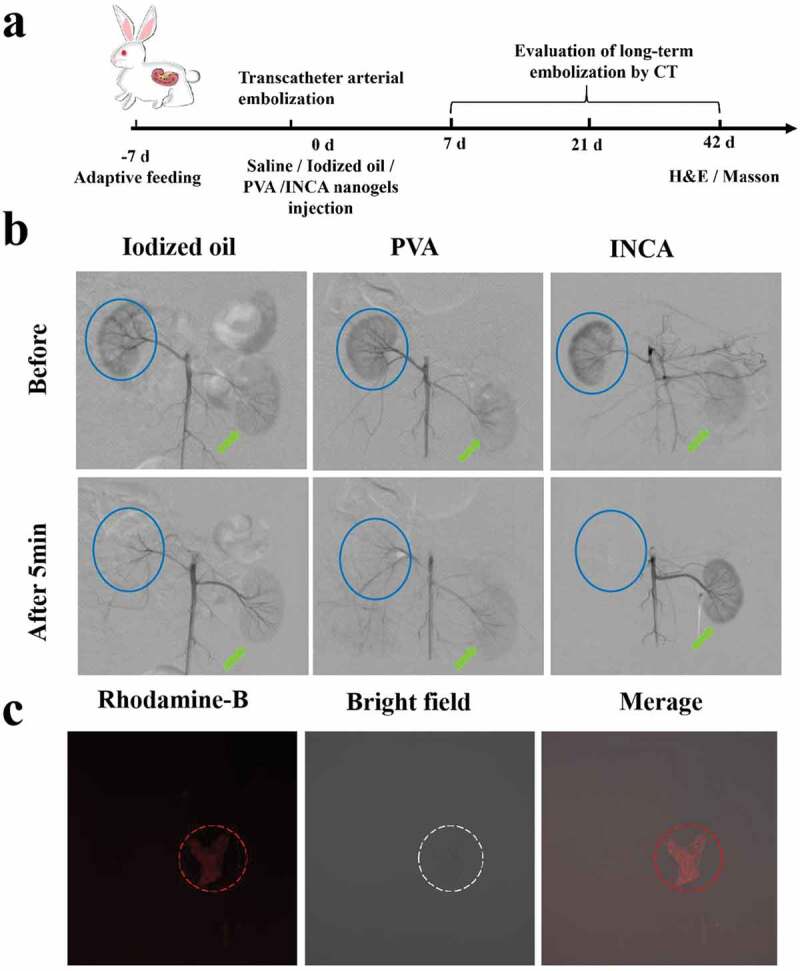
Evaluation of the embolization effect of INCA nanogels. (a) Scheme of the evaluation flow chart of the New Zealand rabbit renal artery embolization model. (b) DSA images of each embolizing agent before and 10 min after embolization, the solid blue circle is the right kidney, and the green arrow is the left kidney. (c) Vascular distribution of INCA nanogels in right rabbit kidney (original magnification, ×200).

The right renal artery of normal rabbits was used to evaluate the effect of embolization because the kidney has abundant vascular branches, which is conducive to accurately evaluating vascular embolism at all levels (Figure 4a). As shown in the DSA profile, the embolization of renal vessels by iodized oil can reach the renal aorta, interlobular artery, and arcuate artery, while PVA can only reach the renal aorta due to its large size, and thus, the embolization effect of renal vessels was poor. INCA nanogels had good liquidity, low viscosity, and were easier to reach the distal renal cortex, compared with other experimental groups, and could achieve comprehensive embolization of the right renal aorta, interlobular artery, and even bulbar arteriole in rabbits. In addition, the cast embolization effect of iodized oil, PVA, and INCA nanogels on rabbit kidneys was evaluated further (Table 1 and Figure 4b). Iodized oil, PVA, and INCA nanogels (1.5 mL) were injected into the right kidney of rabbits in each experimental group. Lidoxanol was administered 5 min later, and partial recanalization of the right kidney was observed in the iodized oil group. At the same time, PVA particles could not completely embolize the right kidney due to their particle size limitation, so part of the contrast agent could enter the right renal artery.

**Table 1.** Embolization effect evaluation of TAE therapy.

Embolization agents	Injection Rate (mL/min)	Dosage(mL)	Embolization effect
Iodized Oil	1	1.5	Recurred within 7 days
PVA	1	1.5	Partial recurred within 21 days
INCA	1	1.5	Long-term embolization lasted for 42 days

**Figure 5.**
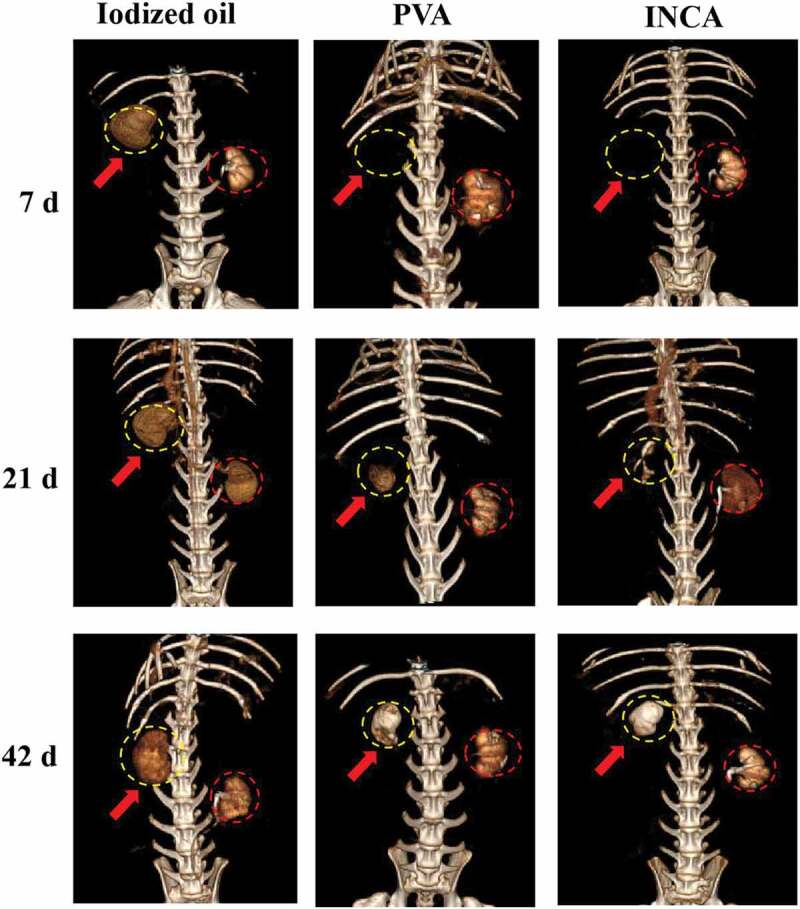
Three-dimensional reconstructed CT images of iodized oil, PVA, and INCA nanogels in the right kidney of rabbits 7 d, 21 d, and 42 d after embolization. The yellow dotted circle and the red arrow point to the right kidney of the rabbit, and the red dotted line is the left kidney of the rabbit.

Interestingly, INCA nanogels showed excellent casting embolization effect on the right kidney of rabbits, and the lumen of the blood vessels of the right kidney was completely blocked in 5 min. Meanwhile, the blood vessels of the left kidney of rabbits showed a sharp contrast to the embolization of the right kidney in all groups. The femoral artery incisions of the experimental rabbits in each group were sutured, and three days later, penicillin sodium and gentamicin sulfate were injected, along with continued feeding. According to coronal CT imaging, follow-up monitoring was performed on the 7th, 21st, and 42nd days (Figure 5 and Figure S2). The iodized oil group had poor embolization of the right kidney on the 7th day after embolization. Lidoxanol (3 mL) was injected intravenously through the rabbit ear margin again, which could easily enter the right renal vessel and imaging. Partial visualization of the right kidney was observed at 21 d and 42 d in the PVA group, and the embolization effect was slightly better than that of the normal saline and iodized oil group. INCA nanogels showed no recurrence of the right kidney on day 7 and day 21, and the volume of the right kidney was significantly smaller than that of the left on day 42, which was considered to be local imaging caused by necrosis and calcification of the right kidney.

INCA nanogels stained with rhodamine-B were also injected into the rabbit’s right renal artery of the rabbit, and then the kidney tissues were sacrificed for frozen sections. The distribution position of the nanogels was determined by fluorescence confocal microscopy. INCA nanogels were evenly distributed in the lumen of the renal vessels without extravascular leakage, which could block the blood vessels at all levels of the right kidney (Figure 4c). Three-dimensional reconstructed CT images verified the above statement (Figure 5). In general, the embolization of INCA nanogels in rabbits’ right kidneys could cause evident right renal insufficiency, resulting in ischemia, necrosis, and atrophy, while the left renal function was unaffected. Overall, INCA nanogels had the most stable effect on casting embolization of the right kidney of rabbits, and the embolization lasted for a long, thus preventing vascular recanalization from occurring easily.

### 3.5. Histopathological evaluation of embolization effect

**Figure 6.**
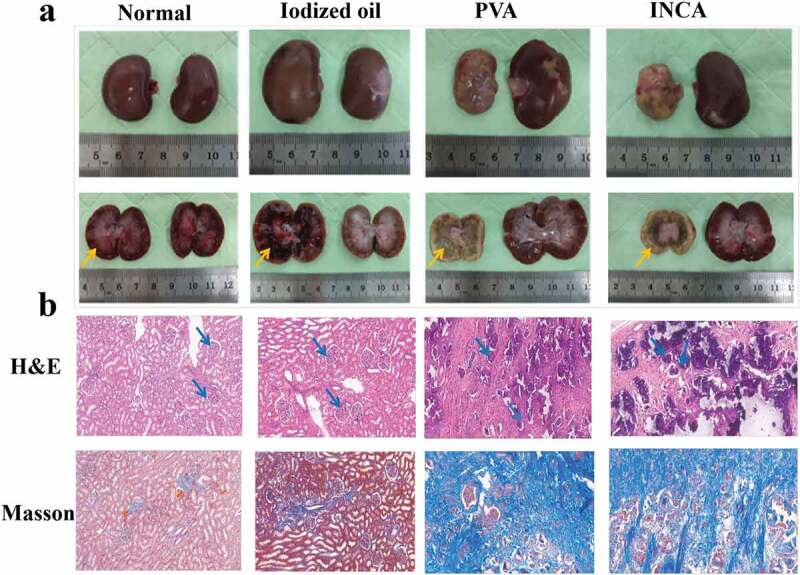
Histopathological evaluation of embolization effect. (a) the Normal physiological, iodized oil, PVA, and the INCA nanogels groups kidney tissue image of 42 days. (b) H&E and Masson staining of the Normal, iodized oil, PVA, and the INCA nanogels groups (original magnification, ×200).

H&E and Masson staining were used to evaluate the pathological differences between embolized and normal renal tissues after 42 days of embolization to further compare the embolization effect of embolization materials in each experimental group. As shown in Figure 6a, each experimental group’s renal tissue gross map shows that the right kidney of the PVA and INCA nanogels groups had evident ischemia, atrophy, and calcification. The results of H&E and Masson staining showed that the glomeruli and tissue margins were unclear, but there were no noticeable pathological changes in normal saline and iodized oil groups (Figure 6b).

### 3.6. Biocompatibility evaluation of INCA nanogels

**Figure 7.**
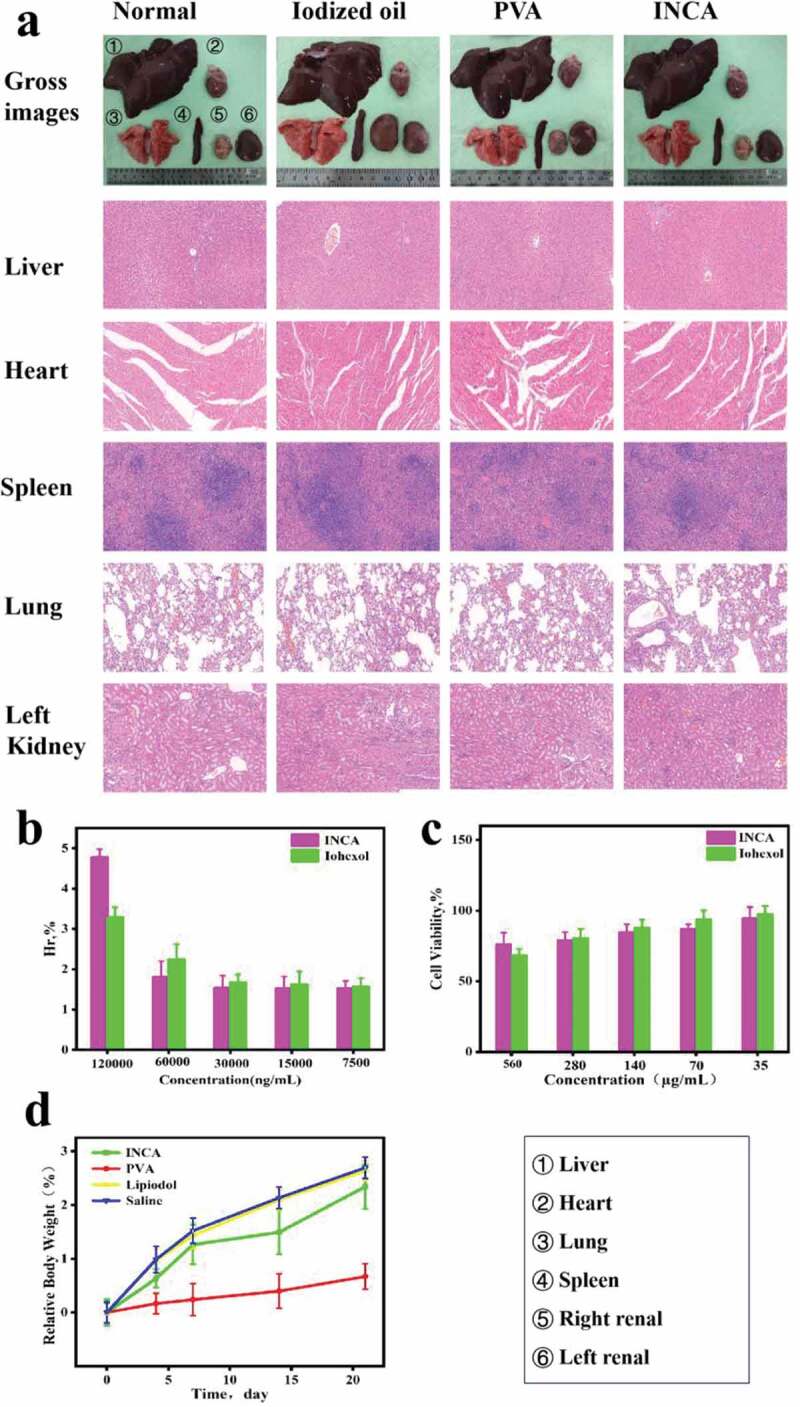
Biocompatibility evaluation of INCA nanogels. (a) Gross images and H&E staining of heart, liver, spleen, lung, and kidney tissues in the normal, iodized oil, PVA, and INCA nanogels groups (original magnification, ×200). (b) The hemolysis rates of normal rabbit blood cells were determined by the concentration dilution of INCA nanogels and iohexol solution to 120 µg/mL, 60 µg/mL, 30 µg/mL, 15 µg/mL, and 7.5 µg/mL, respectively. (c) The survival rate of HepG2 cells was analyzed by INCA nanogels and iohexol solution at 560 µg/mL, 280 µg/mL, 140 µg/mL, 70 µg/mL, and 35 µg/mL. (d) Rabbits’ relative body mass index in the normal, iodized oil, PVA, and INCA nanogels groups.

The ideal embolization material should not only have a sustained and stable embolization effect but also pay attention to the biocompatibility of the material to the body to ensure non-toxic and safe use. Therefore, rabbits’ heart, liver, spleen, lung, and kidney tissues were collected in each experimental group for pathological H&E and Masson staining to judge the damage of embolization materials to each target organ from its morphology. As shown in Figure 7a, the tissue structures of the heart, liver, spleen, lung, and kidney in the normal, iodized oil, PVA, and INCA nanogel groups were clear, with no noticeable pathological changes and good biocompatibility. Subsequently, erythrocyte suspension prepared from normal rabbit whole blood was co-incubated with INCA nanogels and iohexol solution, and the hemolysis rate of erythrocyte was investigated in different concentrations. INCA nanogels at 120 µg/mL, 60 µg/mL, 30 µg/mL, 15 µg/mL, and 7.5 µg/mL exhibited hemolysis rates of 4.77 ± 0.207%, 1.81 ± 0.309%, 1.53 ± 0.311%, 1.52 ± 0.297%, and 1.51 ± 0.182%, respectively. All groups had hemolysis rates of less than 5%, indicating good biocompatibility, and the iohexol solution had similar low hemolysis rates (Figure 7b). HepG2 cells at logarithmic growth were co-incubation with INCA nanogels dispersion and iohexol solution at dilutions of 560 µg/mL, 280 µg/mL, 140 µg/mL, 70 µg/mL, and 35 µg/mL, and the corresponding cell viability of INCA nanogels dispersion was 76.34 ± 8.064%, 79.45 ± 5.286%, 84.93 ± 5.338%, 87.30 ± 3.129%, 94.70 ± 7.826%. At the same time, the cytotoxicity of the iohexol solution was also lower (Figure 7c). In addition, the relative body mass index of rabbits in the experimental group was also measured. The relative body mass index of rabbits in each experimental group did not fluctuate significantly but showed an upward trend overall (Figure 7d). In conclusion, INCA nanogels had good biocompatibility and similar low toxicity to the body and no damage to target organs compared with lipiodol and PVA used in clinical transcaster arterial embolization, thus showing excellent prospects for development and application in the medical field.

## 4. Conclusion

In this study, we learned from the traditional emulsion polymerization method and optimized the reaction system efficiently to prepare a double core-shell spherical structure of poly (N-isopropyl acrylamide)-*co*-acrylic acid nanogel. Its small particle size and good dispersibility endowed the aqueous gel dispersion with good fluidity, which was conducive to achieving complete embolization deep into the peripheral blood vessels. In addition, its excellent temperature sensitivity also induced NIPAM-*co*-AA nanogels gelation at about 34°C. Thus, it could effectively perform sol-gel phase transformation at the physiological temperature of the human body and play the function of solution-gel phase transformation well to solidify in situ and cast in the embolization site without being degraded easily.

Iohexol is a common X-ray opaque contrast agent. Iohexol solution (200 mg/mL) was mixed with 7 wt% NIPAM-*co*-AA nanogels to obtain INCA nanogels with radiopaque functions of embolization and intraoperative imaging to further optimize the application advantages of NIPAM-*co*-AA nanogels as a new embolization material. This novel intelligent embolization material showed broad application value compared with the commonly used clinical embolization materials. Presently, iodized oil has limitations, such as fast blood removal and easy recanalization of embolized blood vessels. Besides, PVA has large particles and cannot penetrate small blood vessels, resulting in incomplete embolization. At the same time, it lacks imaging properties that must be combined with iodized oil for repeated embolization, which is time-consuming. As a result, INCA nanogels can effectively solve the current flow-embolization contradiction by addressing the shortcomings of the aforementioned materials. Their good fluidity makes catheter injection easier, and it is difficult to stick to tube blocking, which can monitor the entire embolization process to avoid mis-embolization and leakage. The normal rabbit renal embolization model demonstrated that INCA nanogels could be dispersed more effectively in the distal area of the rabbit’s right renal artery than in other experimental groups. The embolization material was transferred without reflux from the renal cortex to the renal hilus and then to the main renal artery. After 42 days of embolization, the right kidney of the rabbit showed evident ischemic necrosis, atrophy, and calcification, indicating that INCA nanogels can achieve complete embolization on the renal artery. Therefore, this bio-compatible nanogel embolization material has the potential as a new medical embolization material in clinical treatment.

## Abbreviations

TAE: Transcatheter arterial embolization; TACE: Transcatheter arterial chemo-embolization; NIPAM-*co*-AA: poly (N-isopropyl acrylamide)-*co*-acrylic acid nanogel; INCA: Intelligent thermosensitive radiopaque nanogel; DSA: Digital subtraction angiography; NIPAM: N-isopropyl acrylamide; AA: Acrylic acid; AAPH: 2,2’-azobis(2-methylpropionamidine) dihydrochloride; STAC: Octadecyl trimethyl ammonium chloride; MBA: N, N’-methylene bisacrylamide; PVA: polyvinyl alcohol.
